# Making the health system work for the delivery of nutrition interventions

**DOI:** 10.1111/mcn.13056

**Published:** 2020-07-20

**Authors:** Shannon E. King, Talata Sawadogo‐Lewis, Robert E. Black, Timothy Roberton

**Affiliations:** ^1^ Department of International Health Johns Hopkins Bloomberg School of Public Health Baltimore Maryland USA

**Keywords:** health care systems, health integration, maternal and child health, nutrition, nutrition programmes, nutrition specific

## Abstract

Addressing malnutrition requires strategies that are comprehensive and multi‐sectoral. Within a multi‐sectoral approach, the health system is essential to deliver 10 nutrition‐specific interventions, which, if scaled up, could substantially reduce under‐5 deaths in high‐burden countries through improving maternal and child undernutrition. This study identifies the health system components required for the effective delivery of these interventions, highlighting opportunities and challenges for nutrition programmes and policies. We reviewed implementation guidance for each nutrition‐specific intervention, mapping the delivery process for each intervention and determining the health system components required for their delivery. We integrated the components into a single health systems framework for nutrition, illustrating the pathways by which health system components influence household‐level determinants of nutrition and individual‐level health outcomes. Nutrition‐specific interventions are typically delivered in one of four ways: (i) when nutrition interventions are intentionally sought out, (ii) when care is sought for other, unrelated interventions, (iii) at a health facility after active community case finding and referral, and (iv) in the community after active community case finding. A health system enables these processes by providing health services and facilitating care seeking for services, which together require a skilled and motivated health workforce, an effective supply chain, demand for services and access to services. The nutrition community should consider the processes by which nutrition‐specific interventions are delivered and the health system components required for their success. Programmes should encourage the delivery of nutrition interventions at every client–provider interaction and should actively generate demand for services—in general, and for nutrition services specifically.

**Key messages**

- The health system plays an essential role as the primary delivery platform for nutrition‐specific interventions that reduce maternal and child malnutrition. Efforts to improve nutrition should include investments in and strengthening of the health system.
- Understanding the processes within the health system that contribute to the delivery of these nutrition‐specific interventions is necessary to improve coverage of these interventions by identifying bottlenecks to the delivery and uptake of services.
- The nutrition community should consider the processes and health system components required for successful nutrition programmes, namely, a skilled and motivated health workforce, an effective supply chain, demand for services and access to services.

## 1 INTRODUCTION

Malnutrition is a complex issue requiring coordinated efforts across sectors to address its underlying causes. The multi‐sectoral nature of nutrition is increasingly being recognized, with most global and national initiatives now including representation from diverse actors and ministerial departments, including health, education, food and agriculture and social protection (Banking on Nutrition Partnership, 2017; Bezanson & Isenman, 2010; Garrett, Bassett, & Levinson, 2011; Gillespie et al., 2013; Reinhardt & Fanzo, 2014; The World Bank, 2013; World Health Organization, 2018). A comprehensive approach to addressing malnutrition requires the engagement of all sectors to deliver both nutrition‐specific interventions (i.e., those that directly address nutritional status) and nutrition‐sensitive interventions (i.e., those that address underlying determinants of nutrition; Banking on Nutrition Partnership, 2017; Ruel et al., 2013; The World Bank, 2013; United Nations Children's Fund, 2015).

The health system plays an essential role in the delivery of nutrition‐specific interventions, particularly those targeted at the well‐being of children and mothers (World Health Organization [WHO], 2019). The 2013 Lancet series on nutrition highlighted 10 nutrition‐specific interventions that, if scaled up, could significantly reduce child mortality associated with undernutrition: folic acid supplementation during pregnancy, multiple micronutrient supplementation during pregnancy, calcium supplementation during pregnancy, balanced energy protein supplementation during pregnancy, exclusive breastfeeding, complementary feeding, vitamin A supplementation (6–59 months), preventive zinc supplementation, management of severe acute malnutrition (SAM), and management of moderate acute malnutrition (MAM; Bhutta et al., 2013). All of these nutrition‐specific interventions are delivered through the health system and specifically address undernutrition issues in mothers and children. While there is substantial evidence for the efficacy and potential impact of nutrition‐specific interventions, less is understood about how to deliver the interventions at scale, much less the role of health systems in facilitating or hindering nutrition programmes (Pérez‐Escamilla & Engmann, 2019). There is increasing emphasis on implementation research in the nutrition community to understand how to deliver interventions with high fidelity, dose and sustainability (Garrett, 2008; Leroy & Menon, 2008; Menon et al., 2014; Paina & Peters, 2012; Pérez‐Escamilla & Engmann, 2019; Salam, Das, & Bhutta, 2019). Successful implementation requires intentional changes to current processes within the existing systems (Damschroder et al., 2009). Fundamentally characterizing and understanding these processes is a necessary precursor to any implementation research.

Beyond the provision of nutrition‐specific interventions, the health sector plays a role in the delivery of nutrition sensitive interventions, (e.g., the provision of reproductive health services to women). We chose to focus the scope of this work on these 10 nutrition‐sensitive interventions for which there is strong evidence of effect. However, many nutrition sensitive interventions delivered through the health system require the same elements.

In this paper, we identify and describe the health system components required for the delivery of nutrition‐specific interventions and explore how the framework could be used to identify opportunities for increasing coverage of nutrition interventions. Understanding the health system as a complex adaptive system that includes path dependence processes, non‐reversible processes that lead to other outcomes, is integral to the increased and sustained coverage of these nutrition interventions (Paina & Peters, 2012). Identifying the components of the health system that are influential to the delivery of programmes is a first step to improving the implementation of those programmes. Our analysis brings together the nutrition literature with the health systems literature to see how ‘upstream’ health system components affect nutrition programmes and ultimately affect population‐level nutrition outcomes. We incorporate the elements of well‐established frameworks (Nixon & Ulmann, 2006; Roberts, Hsiao, Berman, & Reich, 2003; Roemer, 1993; WHO, 2007) and the UNICEF model for malnutrition (Appendix A; UNICEF, 1998) to explicitly highlight the direct linkages between health system components and the delivery processes required for nutrition‐specific interventions.

## 2 METHODS

We reviewed the implementation guidance from major intergovernmental organizations and non‐governmental organizations for each of the 10 nutrition‐specific interventions listed in the 2013 Lancet series on nutrition (Bhutta et al., 2013). For nine of the 10 interventions, we extracted information on the nature of the intervention (the provision of drugs/supplements or counselling), the delivery level (facility or community), the workforce cadre responsible for the intervention (doctor, nurse or occasional trained provider/peer) and the required supplies (drugs/supplements or counselling materials). Due to differences in the interventions highlighted in the Lancet series and the WHO formal implementation guidelines, preventative zinc was included as the provision of a multiple micronutrient powder (MNP). Periconceptual folic acid was not included as it is predominately delivered through fortified foods, which are not within the health system and thus excluded from this analysis. Multiple micronutrient supplements (MMS) are not recommended by WHO for universal use in pregnancy; however, the guidelines for prenatal supplementation put forward that countries may considering substituting MMS for iron folic acid (IFA) when there is evidence of multiple micronutrient deficiencies in women of reproductive age (Bourassa et al., 2019; WHO, 2016). As the provision of MMS is similar to that of IFA, we used the IFA implementation guidelines for MMS.

Using the information extracted from the implementation guidelines, we developed flow diagrams illustrating the delivery process for each intervention: either the steps undertaken by a community member to seek out the intervention or by health system actors to identify community members needing care. We examined commonalities across the flow diagrams and synthesized the results into a set of four unique delivery processes. For each delivery process, we identified the health system service components needed for the process to be successful, including the physical elements required at a health facility (commodities and human resources) and the activities required to promote care seeking or active case finding so that a community member receives the intervention when needed. This led to the development of four service component diagrams depicting the health system components required for the delivery process. Finally, we integrated the service components into a single health systems framework for nutrition. We used evidence from both the health systems literature and nutrition literature to identify the ‘upstream’ building blocks required for each ‘downstream’ service component. Using an iterative process, we expanded the health systems framework to include all components required to deliver the 10 nutrition‐specific interventions.

### 2.1 Ethical considerations

This study did not involve any human subjects research.

## 3 RESULTS

### 3.1 The implementation of nutrition‐specific interventions

Table 1 shows the information extracted for the nine nutrition‐specific interventions for which we found implementation guidance at a global level.

**TABLE 1:** Information from implementation guidance for nutrition‐specific interventions

Intervention	Intervention name	Vitamin A supplementation (Micronutrient Initiative, n.d.; WHO, 2011)	Multiple micronutrient supplementation during pregnancy (WHO, 2016)	Maternal balanced energy protein supplementation (WHO, 2016)	Maternal calcium supplementation (WHO, 2018b)	Prenatal and post‐natal breastfeeding promotion (Gilmore & McAuliffe, 2013; WHO, 2009)	Complementary feeding education (or education + supplementation; UNICEF, 2017; WHO, 2009)	Multiple micronutrient powders (containing zinc; WHO, 2016b)	Management of SAM (USAID, 2019; WHO, 2013; WHO, WFP, UNSSCN, UNICEF, 2007)	Management of MAM (USAID, 2019; WHO, 2012)
	Provision of drug/supplement	Children 6–59 months receive two doses per year with 4–6 months spacing between dose.	WHO: Countries to decide on implementation based on degree of micronutrient deficiencies	Balanced protein energy supplementation (protein less than 35% of the total energy content) in areas of high prevalence of undernutrition	Daily calcium supplement (1.5–2.0 g oral elemental calcium) for populations with low dietary calcium intake. Divide daily dosage into three doses taken at mealtimes.		Appropriate dietary supplementation	In populations where anaemia is a public health problem, 90 sachets of point‐of‐use multiple micronutrient powder should be consumed over a 6‐month period. Composition of MNP differs by age of child: MNP for children 6–23 months should contain 10–12.5 mg of elemental iron; 300 μg of retinol, 5 mg of elemental zinc, with or without other micronutrients to achieve 100% of recommended nutrient intake. MNP for children 2–12 years should contain: 10–12.5 mg of elemental iron (2–4 years) or 12.5–30 mg elemental iron (5–12 years); 300‐μg retinol, 5 mg of elemental zinc, with or without other micronutrients to achieve 100% of recommended nutrient intake.	Early detection for children 6–59 months with MUAC < 11 mm or any degree of bilateral; pitting oedema or WHZ < −3	Nutrient‐dense supplementary food using locally available nutrient dense foods if possible (animal sourced or high quality protein rich plant‐source foods). Foods should meet the nutritional standards specified to meet their needs for weight and height gain and functional recovery. If required, then micronutrient supplements or fortified foods can be added to food.Supplementary foods may be used if local foods are not sufficiently available (protein energy biscuits, LNS and RUSF).
									Inpatient treatment: Provide with a course of antibiotics, RUTF after being stabilized with F‐75 or F‐100 if required, rehydration solution (ORS/ReSoMal/Darrow's solution/Ringer's lactat solution), ART if HIV+, daily high dose vitamin A (if not receiving F‐75, F1002 or RTUF). Outpatient treatment: Provide with a course of antibiotics, RUTF and ART if HIV+	
		Children 6–11 months receive 100,000 IU.	Lancet suggestion: UNICEF UNIMAP formulation (contains recommended dietary allowance of 15 vitamins and minerals including iron and folic acid)							
		Children 12 to 59 months of age receive 200,000 IU.								
	Counselling	On potential side effects, benefits of vitamin A, when to return for next dose	Effective communication about diet and healthy. Communication strategies required to improve adherence and acceptability of supplementation. Strategies for reminding women to take daily supplements and manage side effects	Counselling on the need to increase maternal energy and protein intake	Dietary counselling to promote adequate calcium intake through locally available, calcium‐rich foods	Promotion to support optimal breastfeeding (early, exclusive for 6 months, continued for 2 years)	Promotion to support the introduction of safe and nutritionally adequate complementary foods at 6 months with continued breastfeeding until 2 years	Behaviour change strategies to promote awareness and correct use of the MNP product, hygienic and correct preparation, feeding of complementary foods for young children, healthy diets for older children, breastfeeding practices, hand‐washing with soap, attention to fever in malaria settings and diarrhoea management	Counselling and support for optimal infant and young child feeding based on general IYCF guidelines. Counselling on importance of adherence to medication	Counselling on breastfeeding promotion and support; Education and nutrition counselling for family and other activities that prevent the underlying cause of malnutrition
Delivery level	Facility	Implemented in conjunction with expanded programme on immunization	ANC care	ANC care might require extra visits to be scheduled if ANC visits are not sufficient	ANC care	ANC care	ANC care	Distribution strategies and platforms (e.g., schools and health facility) vary by context depending on what is best suited to reach the target population to ensure uninterrupted supply.	Facility‐based management for initial treatment followed by community management of acute malnutrition (CMAM)	
						Post‐natal care	Post‐natal care			
							Immunization contacts/growth assessment			
						Immunization contacts/growth assessment	Sick child visits/follow‐up			
						Sick child visits/follow‐up				
	Community	Child health events (biannual days)	Outreach ANC care		Task shift to CHWs to ensure community provision of calcium supplements to vulnerable populations	CHW home‐visits	CHW home‐visits	Distribution strategies and platforms (e.g., schools and health facility) vary by context depending on what is best suited to reach the target population to ensure uninterrupted supply.	Utilized predominately in emergency settings	CMAM‐ screened and identified within the community then provided with supplementary food (for RUSF or fortified blend)
									CMAM‐ screened and identified within the community then brought to the facility for initial evaluation and treatment	
Cadre responsible	Doctors/nurse	Provides in conjunction with EPI; could be contributing to child health weeks	Facility‐based ANC services	Facility‐based ANC services	Facility‐based ANC services	Provision of counselling and support in post‐natal care	Provision of counselling and support in post‐natal care	Depending on distribution mechanism, doctors/nurses could provide the product and counselling at a facility‐based visit.	In‐patient facilities	
	Community health workers (CHWs)	Could be contributing to child health weeks	Through community based ANC services		Through community based on task‐sharing	Home‐based CHW counselling visits	Home‐based CHW counselling visits	Depending on distribution mechanism, community health workers could provide the product and counselling when it is delivered through community‐based platforms. Household visits to reinforce the counselling messaging	Community facilities/assessment during any other care provision CMAM for screening and treatment	Community facilities/assessment during any other care provision CMAM for screening
	Occasional trained providers/peers	Trained providers for child health weeks;need to account for travel and displacement of these workers for the child health weeks				Home‐based peer counselling has worked within some contexts; mobilization of community groups	Complementary food supplements	Depending on distribution mechanism, trained providers/peers could provide the product and counselling when it is delivered through community‐based platforms. Household visits to reinforce the counselling messaging		
Supplies	Drugs/supplements	Vitamin A capsules, scissors/nail clippers and waste baskets	Multiple micronutrient supplement	Balanced energy and protein food supplements	Calcium supplements			Multiple micronutrient powders	Antibiotics, RUTF, F‐75 or F‐100, rehydration solution (ORS/ReSoMal/Darrow's solution/Ringer's lactat solution), vitamin A and ART if HIV+ population	Locally available supplementary foods
										Supplementary foods (micronutrient supplements, protein energy biscuits, LNS and RUTF)
		Have 5–10% extra for all supplies								
		Tally sheets, reporting forms								
	Counselling materials	^a^	^a^	^a^	^a^	Tailored IEC materials to the context (values, beliefs and practices)	Tailored IEC materials to the context (values, beliefs and practices)	^a^	^a^	^a^

^a^ Behaviour change materials have not been included within implementation guidelines however programmes frequently include a counselling component with some sort of job aids within service provision to improve the uptake and adherence to the intervention.

**FIGURE 1:**
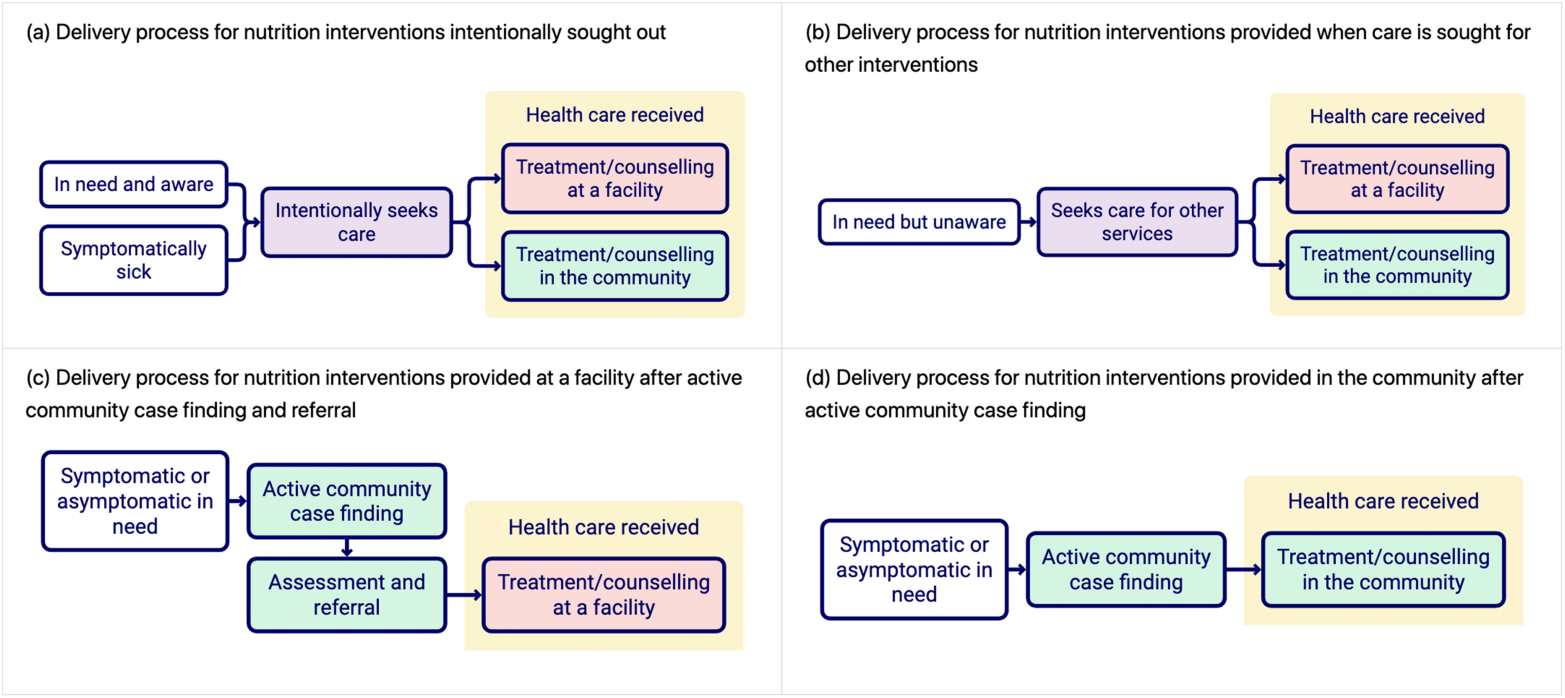
(a–d) The four delivery processes by which an individual may receive one of nine nutrition‐specific interventions. The interventions include vitamin A supplementation, multiple micronutrient supplementation during pregnancy, maternal balanced energy protein supplementation, maternal calcium supplementation, breastfeeding promotion, complementary feeding education (or education + supplementation), multiple micronutrient powders (containing preventative zinc) and management of SAM and MAM

Figure 1 shows the four delivery processes involved in delivering the interventions in Table 1, starting from the initial awareness of one's need for the intervention through to care seeking and receipt of the intervention at the facility or in the community. Figure 1a depicts the delivery process for interventions where individuals explicitly go to a facility or community provider to obtain the intervention; for example, during a vitamin A supplementation campaign, when mothers intentionally bring their children to the vaccination station to receive vitamin A. Figure 1b shows the second delivery process, in which an individual seeks care for an unrelated health reason and is offered the nutritional intervention during that care visit, for example, when a mother brings a child to a provider to treat fever and the provider gives the child vitamin A supplementation because the child had not received it during a campaign. The third and fourth delivery process (Figure 1c,d) involve active case finding in the community to find individuals requiring an intervention. In Figure 1c, a community health worker identifies children in need of a service and refers those children to a facility for care, as typically occurs in SAM programmes. In Figure 1d, the community health worker provides the care directly themselves, for example, in community‐based management of MAM.

Each of the nutrition‐specific interventions is delivered through at least one of the delivery processes (Figure 1a–d); however, depending on the context, it can be delivered through more than one, as shown in Table 2.

**TABLE 2:** Provision of the nutrition interventions through the four delivery processes

	Nutrition interventions intentionally sought out	Nutrition interventions that are provided through other care seeking intentions	Nutrition interventions delivered through facility care after active community case finding	Nutrition interventions delivered within the community after active community case finding
Vitamin A supplementation	X	X		
Multiple micronutrient supplementation during pregnancy	X	X		
Maternal balanced energy protein supplementation		X		
Maternal calcium supplementation		X		
Breastfeeding promotion		X		
Complementary feeding education (or education + supplementation)		X		
Multiple micronutrient powders (containing preventative zinc)		X		X
Management of SAM	X	X	X	X
Management of MAM	X	X		X

### 3.2 How the health system delivers nutrition‐specific interventions

**FIGURE 2:**
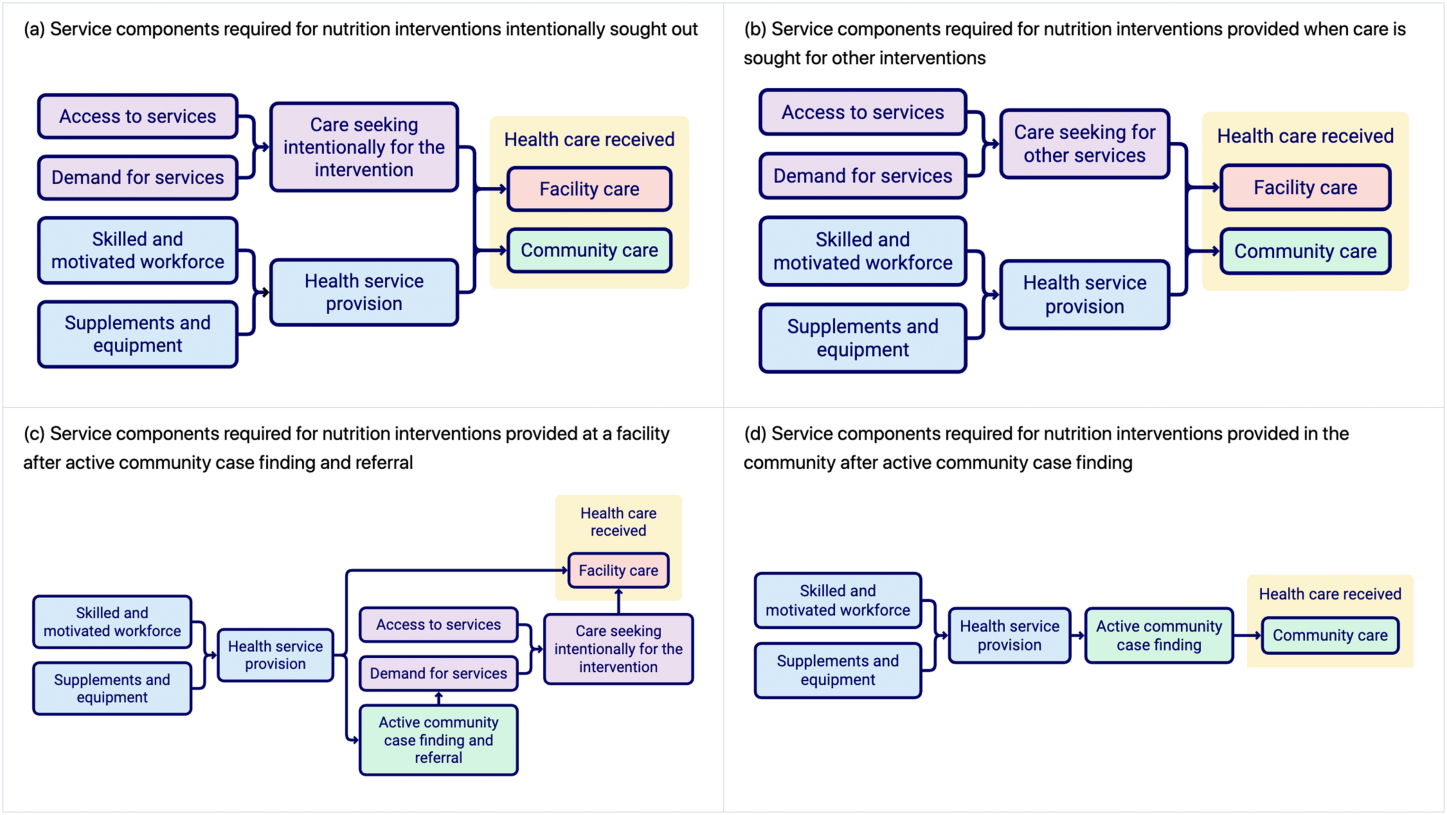
(a–d) The components of the health system that are necessary to deliver nutrition‐specific interventions through each of the four delivery processes in Figure 1a–d

We overlayed information from the implementation guidance documents onto the four delivery processes to create four complementary diagrams of service components required to achieve intervention coverage (Figure 2a–d).

These service component diagrams highlight two pathways by which health systems contribute to intervention delivery: first, by *providing health services* and, second, by *promoting and facilitating care seeking* for health services (either care seeking for specific services, or care seeking in general). These pathways, in turn, depend on four common health systems components:

1. A skilled and motivated health workforce

Health workers are needed for both *providing health services* and for *promoting and facilitating care seeking.* The delivery of all nutrition‐specific interventions requires a direct interaction between a health worker and a patient (if we consider folic acid supplementation in lieu of fortification). Facility‐based interactions are necessary both for direct service provision and for promoting care seeking for future services. Community health workers (CHWs) are needed for active case finding at village level, without which, demand and care seeking for certain interventions (for example, MAM and SAM), would likely be lower than current levels. Further, quality of the intervention delivered, with particular emphasis on those requiring counselling (for example, breastfeeding and complementary feeding promotion), depends on the skills and motivation of the health workforce.

- An effective supply chain

A second component needed for *providing health services* is a well‐functioning supply chain, to make available the necessary drugs or supplements needed for intervention delivery. Vitamin A, MMS, balanced energy, maternal calcium supplementation and management of SAM and MAM each require either a nutrition supplement or a ready‐to‐use therapeutic food (RUTF). The availability of these products is contingent on the supply chain moving the products from a central purchasing unit or storage facility to individual health facilities, local retailers and CHWs. Stockouts are a barrier to care and can diminish subsequent care seeking by community members.

- Demand for services

Six of the eight nutrition‐specific interventions in Table 2 require community members to seek care from a provider. Generating demand for health services is critical to improving coverage of these interventions. The health system can play a role in *promoting care seeking*, through health education, messaging campaigns and direct counselling of patients who come to facilities for unrelated services. Community health workers, when appropriately trained, equipped and supported, can bolster demand generation at the village or community level.

- Access to services

Care seeking is a product not only of an individual's *willingness* to seek care but also of their *ability* to seek care. Various constraints can hinder care‐seeking ability, including poor geographical access, financial barriers and restrictive socio‐cultural forces. The health system plays an essential role in *promoting and facilitating care seeking* by ensuring that there are adequate numbers of well‐positioned health facilities, by adopting close‐to‐community strategies whenever possible, and through financing strategies that enable all community members to access care when needed.

**FIGURE 3:**
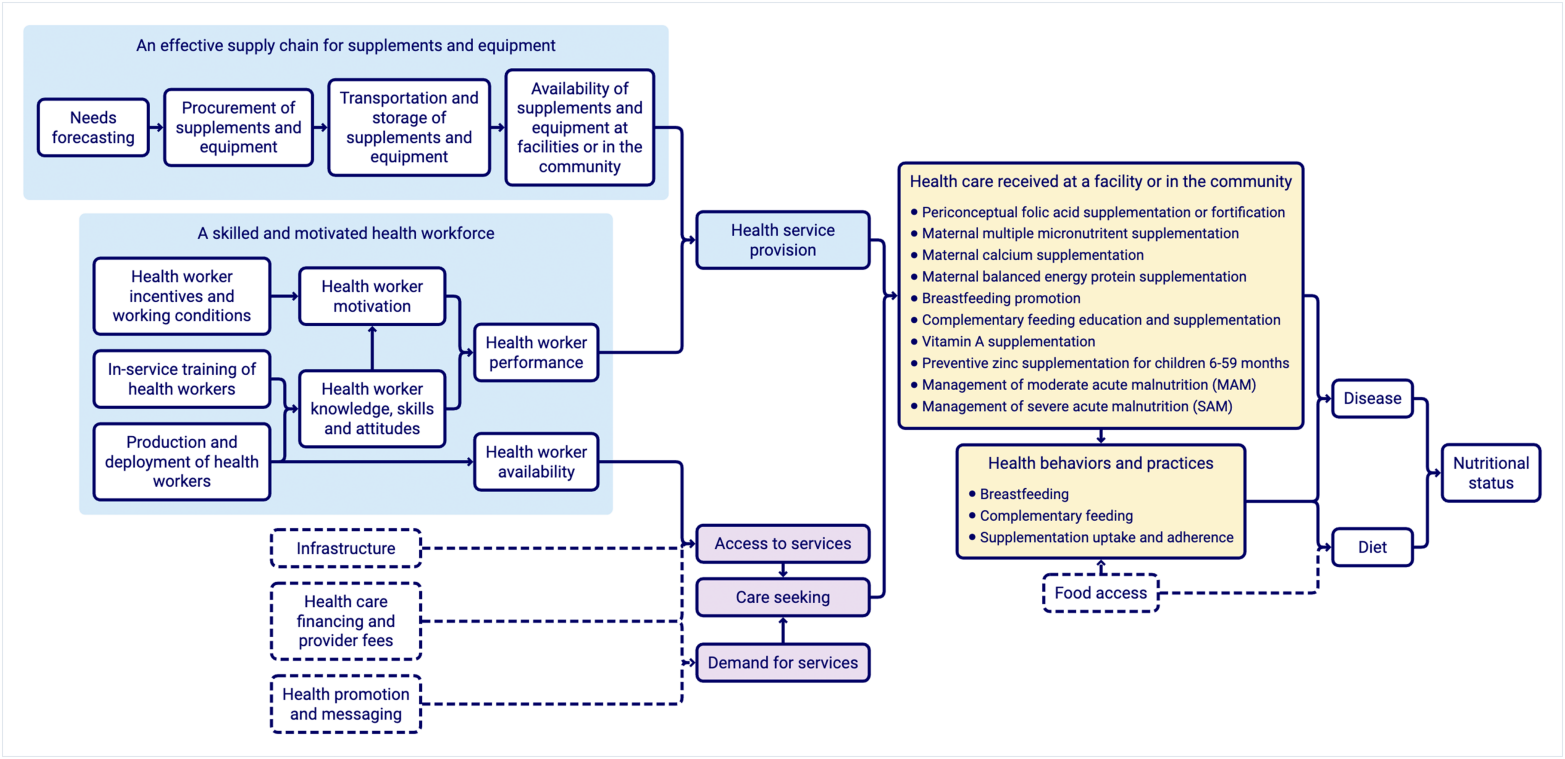
Presents a conceptual framework of the health system demonstrating how components of the health system influence the delivery of the nutrition‐specific interventions and, in turn, nutrition

Figure 3 represents a health systems framework for nutrition, generated by integrating and expanding the four service component frameworks. It shows the path dependency of the system; how the ‘upstream’ health system components affect the ‘downstream’ nutritional status of individuals and population‐level health outcomes. While the framework ends with health outcomes, nutritional status has an intergenerational effect contributing to child development, productivity and human capital.

## 4 DISCUSSION

A strong health system is essential for the delivery of nutrition‐specific interventions (WHO, 2019). Many nutrition programmes fail to anticipate health system bottlenecks, resulting in poor implementation strength and low population coverage (Jennings, Gillespie, Mason, Lotfi, & Scialfa, 1991; Neufeld, Baker, Garrett, & Haddad, 2017; Pérez‐Escamilla & Engmann, 2019). There is much evidence for the efficacy of nutrition interventions, but there is less evidence on the implementation of nutrition programmes at scale to achieve meaningful impact (Garrett, 2008; Leroy & Menon, 2008; Weber, Galasso, & Fernald, 2019). To increase coverage of nutrition interventions, we should consider opportunities to strengthen key health system components. A health systems framework, such as Figure 3, can help to illuminate the upstream factors and causal pathways that affect the delivery of nutrition interventions. Understanding these health system components—and how they vary in different country contexts—can help to explain programme successes and failures and help to identify levers for improvement (Garrett, 2008; Menon et al., 2014; Paina & Peters, 2012). Despite the identification of these areas of opportunity, there still remains gaps in our understanding of how to optimize the effectiveness of these components. For example, to address the existing workforce shortage, efforts such as task‐sharing and the creation of community‐based health worker cadres have been attempted yielding varying degrees of success (Bangdiwala, Fonn, Okoye, & Tollman, 2010; Lehmann, Van Damme, Barten, & Sanders, 2009; Scott et al., 2018).

Future analyses could use implementation research or statistical modelling to identify the health system components that are responsible for the greatest bottlenecks and thus the greatest opportunities for impact. While we should aim to generate more evidence on the implementation of nutrition programmes, we should also draw on the broader health systems literature and literature from other health domains. Lessons could be learned from non‐nutrition interventions that operate through similar delivery mechanisms.

The four identified health systems components reflect immediate outputs of the health system; however, it is important to note that health systems literature highlights many other elements beyond the four highlighted in this paper, necessary for successful provision of care, for example, health information systems, financing or leadership/governance (WHO, 2007). More broadly speaking, we recognize the need for investments and efforts to strengthen the overall health system within LMICs.

In addition to strengthening health systems, we should also consider adjusting delivery processes to make better use of existing health systems as they are now (Salam et al., 2019). As seen in Table 2, many nutrition‐specific interventions are currently delivered when a person is seeking care for another reason and then given a nutrition intervention. This highlights two opportunities to increase coverage.

First, programme implementers could work with other health‐sector actors in low‐ and middle‐income countries (LMICs) to increase the number and frequency of interactions—*for any reason*—that people have with the health system, particularly during the critical window of opportunity for malnutrition known as the first 1,000 days, from conception through to 2 years of age. Each interaction with the health system offers an opportunity to review an individual's need for nutrition services (e.g., balanced energy protein supplementation and vitamin A supplementation), to provide counselling on the need to adhere to the nutrition supplementation they have already been given (e.g., maternal calcium supplementation or MMS), or to provide continued support and address current challenges with breastfeeding or complementary feeding. As long as the health worker is skilled and has the material required for the intervention, each interaction with the health system is an opportunity. The relevant strategies here thus include (a) improving demand for, and access to, health services *in general* and (b) enhanced pre‐service and in‐service training of health workers to ensure they use every patient interaction to assess, treat and counsel on nutrition‐related issues.

Second, for interventions that are currently delivered when people seek care for unrelated services (Figure 1b), programme implementers could initiate a shift in delivery process, to either encourage people to intentionally seek care for the intervention (Figure 1a) or to adopt a community‐based, active‐case‐finding strategy for the intervention (Figure 1c or Figure 1d). A health promotion strategy that increases awareness of the need for, and benefits of, nutrition‐specific interventions could increase both the delivery of interventions, the timeliness of reception of nutrition care and adherence to nutrition‐impacting behaviours. Although we should not overburden CHWs, it seems feasible that CHWs might promote or refer children for an additional set of nutrition services. Some nutrition products that are currently only available at facilities might also be made available and administered at village level. Programmes may already be adopting these approaches; in which case, we should bring global guidelines into line with what programmes are actually doing in practice.

Lastly, while the health system plays an important role in the delivery of nutrition‐specific interventions, it is only one of the sectors that should be considered within a multi‐sectoral strategy to address malnutrition (Appendix B). Education, social protection, food and agriculture and WASH all play an important role in supporting the delivery of both nutrition‐specific and nutrition‐sensitive programmes. A similar analysis could be conducted for each of these sectors to understand the roles they play in the delivery of nutrition interventions.

## 5 CONCLUSION

Nutrition is a multi‐sectoral problem, but for nutrition‐specific interventions, the health sector is critical. The nutrition community should consider the processes by which nutrition‐specific interventions are delivered and the health system components required for their success, namely, a skilled and motivated health workforce, an effective supply chain, demand for services and access to services. A strong health system will both provide health services *and* promote and facilitate care seeking for those services. Programmes should encourage the delivery of nutrition interventions at every client–provider interaction and should actively generate demand for services—in general, and for nutrition services specifically. To reach an entire population, an active community workforce as part of the health system is likely needed for all nutrition interventions.

## CONFLICTS OF INTEREST

The authors declare that they have no conflict of interest.

## CONTRIBUTIONS

SEK, TR and TSL performed the research. RB contributed essential insights and experiences to the work. SEK and TR drafted the first version of the manuscript. All contributed to multiple revisions of the manuscript and read and approved the final version submitted.

## APPENDIX A THE UNICEF CONCEPTUAL FRAMEWORK OF UNDERNUTRITION

Appendix A presents the UNICEF model for malnutrition (UNICEF, 2013) that is the basis for understanding the determinants of malnutrition.

## APPENDIX B MULTI‐SECTORAL FRAMEWORK FOR NUTRITION

Appendix B presents a multi‐sectoral framework for nutrition emphasizing the relevant in addressing malnutrition.
